# Problematic diagnosis of substance-induced disorders in ICD-11

**DOI:** 10.1192/j.eurpsy.2024.1763

**Published:** 2024-09-18

**Authors:** Jørgen G. Bramness, Carsten Hjorthøj, Solja Niemelä, Heidi Taipale, Eline B. Rognli

**Affiliations:** 1 Institute for Clinical Medicine, UiT The Arctic University of Norway, Tromsø, Norway; 2 Section for Clinical Addiction Research, Oslo University Hospital, Oslo, Norway; 3 National Advisory Unit on Concurrent Substance Abuse and Mental Health Disorders, Innlandet Hospital Trust, Brumunddal, Norway; 4 Department of Drugs and Tobacco, Norwegian Institute of Public Health, Oslo, Norway; 5 Copenhagen Research Center for Mental Health – CORE, Mental Health Centre Copenhagen, Copenhagen, Denmark; 6 Section of Epidemiology, Department of Public Health, University of Copenhagen, Copenhagen, Denmark; 7 Department of Psychiatry, University of Turku, Turku, Finland; 8 Addiction Psychiatry Unit, Department of Psychiatry, Turku University Hospital, Turku, Finland; 9 Niuvanniemi Hospital, Kuopio, Finland; 10 Department of Clinical Neuroscience, Karolinska Institutet, Stockholm, Sweden; 11 Center for Psychiatry Research, Stockholm City Council, Stockholm, Sweden

## Abstract

The ICD-11 was introduced in January 2022. In chapter 6, “Mental, behavioral and neurodevelopmental disorders” we find the section “Disorders due to substance use and addictive behaviors” (section 6C4). Changes from the ICD-10 in this section include broadening the categories of harmful use and dependence, including more types of substances, and the addition of more behavioral addictions (gaming disorder). These changes have been discussed and debated [1].

The ICD-11 was introduced in January 2022. In chapter 6, “Mental, behavioral and neurodevelopmental disorders” we find the section “Disorders due to substance use and addictive behaviors” (section 6C4). Changes from the ICD-10 in this section include broadening the categories of harmful use and dependence, including more types of substances, and the addition of more behavioral addictions (gaming disorder). These changes have been discussed and debated [1].

The fact that ICD-11 has continued and expanded on ICD-10 in diagnosing *secondary* substance-induced mental disorders has received less attention. While ICD-10 included substance-induced psychosis (F1x.5; ICD-11: 6C4x.60-62), ICD-11 also includes substance-induced mood disorders (6C4x.70), anxiety disorders (6C4x.71), obsessive-compulsive and related disorders (6C4x.72) and impulse control disorders (6C4x.73). We have previously shown that a large proportion of those with substance-induced psychosis are later diagnosed with primary psychotic disorders such as schizophrenia and bipolar disorder [2, 3], and the causal complexity as well as the nosologic validity of the diagnosis may be discussed [4, 5]. We believe the expansion with even more substance-induced diagnoses is problematic and would like to comment.

Section 6C4 includes “Disorder due to substance use and addictive behaviors [that] are mental and behavioral disorders that develop as a result of the use if predominantly psychoactive substances including medications, or specific rewarding or reinforcing behaviors” [6]. We believe that this indicates causal inference that is adequate for diagnoses like harmful substance use (6C4x.1) or substance dependence (6C4x.2), but for the above-mentioned secondary mental disorders, we believe it to lean on assumptions that are not fulfilled. A pure pharmacological effect of the substances can of course be seen in the acute intoxication phase or during withdrawal, causing short-term anxiety or even psychotic symptoms, but this is not what is meant by these diagnoses. The codebook clearly states that a substance-induced mental disorder can be assigned when symptoms develop during or soon after intoxication or withdrawal or discontinuation, given that the specific substance can produce such symptoms, and where the duration or intensity of the symptoms of mental disorders are in *substantial excess* of symptoms characteristic of intoxication or withdrawal.

For most mental disorders we do not know the actual underlying cause. This may be due to a lack of knowledge, but also because these disorders are complex with an intricate interplay between social, biological, and psychological factors. This is the major reason why modern-day diagnostic systems (since DSM-III) avoid including causal mention in the diagnoses [7], leaving behind diagnosis like reactive versus endogenous depression or psychosis. One of the major exceptions to this is post-traumatic stress disorder (PTSD). The other major exception is substance-induced mental disorders.

The idea that a temporal relationship is enough to establish that drug use is “the reason” for anxiety, depression or psychosis is a crude simplification of the comprehensive Bradford-Hill criteria for establishing causality [8]. Still, disentanglement of temporality is the major way of ascertaining causality in the diagnostic criteria and diagnostic instruments like the Psychiatric Research Interview for Substance and Mental Disorders (PRISM) [9]. For a primary mental disorder, symptoms must either precede the onset of substance use, persist for a substantial period after cessation of substance use, or the patient must have had a previous episode of the mental disorder without substance use. The mental disorder is assumed to be substance-induced If this cannot be established. This is highly problematic as patients who have used substances for several years, and present with ongoing substance use and symptoms of mental disorders, after such assessments will end up with substance-induced disorders. While we acknowledge the contributing effect of substance use in such cases, we find it problematic that other contributing factors, such as personal disposition, life events, difficult circumstances, and poor coping behavior, are ignored. A substance user may never be diagnosed with a primary disorder as all explanatory weight is placed on the substance use. This is done even if there is little evidence indicating that there is a difference in clinical presentation, course, or indeed in the treatment that should be offered, for example, primary versus secondary depression (the latter following substance use) [10, 11]. Of course, patients may benefit from reducing substance use or abstaining, but this is no different between a primary and a secondary disorder, the former also often using substances. We believe that the only contribution of a division between a primary and secondary disorder is (if any) to give an excuse to deliver less optimal treatment to those with secondary disorders. One could be led to believe that if you just stop taking drugs all will be fine.

With reference to somatic disorders, we know that some of these are 100% attributable to substance use. For example, we speak of alcoholic liver disease and alcoholic cardiomyopathy. But as soon as we know that substance use is only a major contributing factor to the disorder (but not the only cause), we avoid using substances in the diagnosis. We do not say alcoholic hypertension. And in disorders where substances play an even smaller role (but still significant) it would be unthinkable to include substance use in the diagnosis. We would never say alcoholic breast cancer, cannabis-induced asthma, or smoking-induced chronic obstructive pulmonary disease. Just as we do not say stress- or obesity-induced cardiovascular event. This is, at least in part, because we acknowledge that the etiology of these disorders is complex, and the precise role of one risk factor such as substance use is difficult to ascertain. Also, it should not have and *de facto* has no impact on treatment. But coming to substance use and psychiatry all such caution seems to be thrown overboard. We have a feeling that this is due both to disrespect for psychiatric patients and a moralistic view of substance use. There might also be an element of the old psychiatry tradition wanting to avoid treating patients with substance use disorders.

If a patient with anxiety disorder and cannabis use would come into your office and the anxiety disorder has a debut after cannabis use onset, ICD-11 says that the patient should be given a diagnosis of 6C41.71 (cannabis-induced anxiety disorder) and 6C41.10/11 (harmful use of cannabis). We suggest a better way of diagnosing this patient, and consistent with the concept of dual disorders, would be a diagnosis of 6C41.10/11 (harmful use of cannabis) and 6B0x (an anxiety disorder). There is no evidence that cannabis-induced anxiety disorder should be treated any differently than primary anxiety disorder with concurrent cannabis use, and patients with a primary anxiety disorder will probably also benefit from reducing their cannabis use. Ultimately, the clinician cannot “know” that the malady they are dealing with stems from cannabis.

We believe that dual diagnoses are dual diagnoses and should be diagnosed as such. The evidence of drug-induced disorders being completely or for the most part caused by drug use is at best speculative and lacks scientific basis and may lead to less than optimal treatment and a worse outcome for vulnerable patients.
